# Influence of Chronic Ethanol Consumption on Apoptosis and Autophagy Following Transient Focal Cerebral Ischemia in Male Mice

**DOI:** 10.1038/s41598-020-63213-2

**Published:** 2020-04-09

**Authors:** Chun Li, Jiyu Li, Guodong Xu, Hong Sun

**Affiliations:** Department of Cellular Biology & Anatomy, Louisiana State University Health Sciences Center-Shreveport, Shreveport, LA USA

**Keywords:** Stroke, Stroke

## Abstract

Stroke remains one of the leading causes of permanent disability and death worldwide. Apoptosis and autophagy are two key elements involved in ischemic brain damage. Ethanol is a commonly used and abused chemical substance that affects the prognosis of ischemic stroke. We determined the influence of chronic ethanol consumption on apoptosis and autophagy following transient focal cerebral ischemia. Male C57BL/6 J mice were randomly divided into three groups and gavage fed with 0.7 and 2.8 g/kg/day ethanol or volume-matched water daily for 8 weeks. DNA fragmentation, TUNEL-positive neurons, cleaved caspase-3-positive neurons, translocation of mitochondrial cytochrome C and apoptosis inducing factor (AIF), LC3B-positive neurons, and expression of LC3B, Beclin-1 and Bcl-2 in peri-infarct cortex were evaluated at 24 hours of reperfusion after a 90-minute unilateral middle cerebral artery occlusion (MCAO). Cerebral ischemia/reperfusion (I/R) injury was significantly improved in the 0.7 g/kg/d ethanol group but worsened in the 2.8 g/kg/d ethanol group. DNA fragmentation was significantly increased at 24 hours of reperfusion in all groups. However, the magnitude of the increase was significantly less in the 0.7 g/kg/d ethanol group. In addition, both cleaved caspase-3-positive neurons and TUNEL-positive neurons were significantly less in 0.7 g/kg/d ethanol group. Furthermore, translocation of mitochondrial cytochrome C and AIF was significantly alleviated in the 0.7 g/kg/d ethanol group. On the other hand, baseline expression of LC3B was significantly reduced in the 2.8 g/kg/d ethanol group. Post-ischemic expression of LC3B and LC3B-positive neurons were significantly attenuated in both 0.7 and 2.8 g/kg/d ethanol groups. Moreover, although post-ischemic expression of Beclin-1 was not altered in the ethanol groups, post-ischemic expression of Bcl-2 was significantly greater in both 0.7 and 2.8 g/kg/d ethanol groups. Our findings suggest that light ethanol consumption may protect against cerebral I/R injury by suppressing post-ischemic apoptosis, whereas heavy ethanol consumption may exacerbate cerebral I/R injury by suppressing autophagy.

## Introduction

Stroke continues to be one of the main causes of permanent disability and mortality globally^1,2^. Ischemic stroke accounts for about 87 percent of all strokes and happens when an artery supplying blood to the brain gets obstructed. Due to the use of reperfusion/recanalization therapies in acute ischemic stroke, transient focal cerebral ischemia currently becomes a common type of ischemic stroke. Although recanalization/reperfusion is very important for restoring normal blood flow, it can induces and worsens brain damage, named cerebral ischemia/reperfusion (I/R) injury^3^. The mechanisms underlying cerebral I/R injury include many interacting elements, such as oxidative/nitrosative stress, inflammation, apoptosis, and autophagy^3–5^.

Apoptosis contributes to a significant proportion of neuron death in the ischemic penumbra during reperfusion^4^. Apoptosis is a form of programmed cell death and indicated by a series of typical morphological features including formation of apoptotic bodies, chromatin condensation, and cell and nuclear shrinkage. A biochemical hallmark of apoptosis is cleavage of nuclear DNA into internucleosomal fragments. There are three known apoptotic pathways, the perforin/granzyme, extrinsic (death ligand), and intrinsic (mitochondrial) pathways. Caspase-3 serves as a common effector caspase in all three apoptotic pathways^6^. Previous studies have demonstrated that the intrinsic and extrinsic pathways are triggered after cerebral ischemia^7^. On the other hand, autophagy appears a double-edged sword for the survival of neurons following cerebral ischemia^5,8^. Autophagy is considered as a self-eating cellular catabolic process during critical times of development and nutrient stress, through which damaged organelles, aggregated or misfolded proteins, as well as intracellular pathogens are degraded and recycled for the maintenance of cellular homeostasis and normal cellular function^9^. So far three primary types of autophagy, macroautophagy, microautophagy, and chaperone-mediated autophagy, have been reported. The most common and the best-investigated type of autophagy is macroautophagy, which is featured by the formation of autophagosomes. LC3B reflects the number of autophagosomes and thus is currently the most widely used autophagosome marker^10^. Accumulating evidence suggests that macroautophagy is activated following cerebral ischemia^5,8^. However, whether post-ischemic autophagy is beneficial or detrimental is still controversial^8^.

Ethanol is a commonly used and abused chemical substance. Epidemiological studies have found that heavy ethanol consumption increases the morbidity of ischemic stroke and worsens the prognosis of ischemic stroke, whereas mild-moderate ethanol consumption may reduce the morbidity of ischemic stroke and decrease infarct volume and mortality from ischemic stroke^11–16^. Consistently, we recently found that ethanol consumption dose-dependently alters cerebral I/R injury in rodents^17^. Increasing evidence indicates that chronic ethanol consumption alters apoptosis and autophagy in many tissues and organs, such as liver, pancreas, heart, and brain^18–22^. Thus, our goal was to determine whether the influence of ethanol on cerebral I/R injury is associated with an altered apoptosis and/or autophagy.

## Methods

### Animal models of ethanol preconditioning

All of the procedures and protocols were approved by the Institutional Animal Care and Use Committee (IACUC) at the Louisiana State University Health Science Center (LSUHSC)-Shreveport and performed in accordance with the guidelines stated in the National Institutes of Health Guide for the Care and Use Laboratory Animals. Thirty-six male C57/BL6J mice (20–25 g, 10–12 weeks) were randomly divided into three groups: control (n = 12), 0.7 g/kg/d ethanol (n = 12), and 2.8 g/kg/d ethanol (n = 12). Ethanol groups were gavage fed with 10 ml/kg 7% (0.7 g/kg/d ethanol group) or 28% (2.8 g/kg/d ethanol group) ethanol once a day for eight weeks. The control group was gavage fed with 10 ml/kg water. At the end of 8 weeks of feeding, blood pressure, heart rate, and fasting blood glucose were measured similarly as described previously^17^. Blood pressure and heart rate were measured using a CODA mouse tail-cuff system (Kent Scientific, Torrington, CT, USA). Prior to the actual measurement, mice were trained for three consecutive days to acclimate to being restrained and to also having the tail cuff placed on them. Fasting blood glucose was measured by Bayer Breeze2 Blood Glucose Meter (Bayer HealthCare, Mishawaka, IN, USA). Prior to the measurement, mice were fasted for 12 hours during the daytime.

### Transient focal cerebral ischemia

All mice were subjected to transient focal cerebral ischemia, which was induced by unilateral MCAO for 90 minutes as described previously^17^. To avoid a possible effect of acute ethanol, ethanol was not given on the day before the experiment. Prior to the procedure, mice were anesthetized with isoflurane (induction at 5% and maintenance at 1.5%) in a gas mixture containing 30% O_2_/70% N_2_ via a facemask. Body temperature was maintained with a temperature-controlled heating pad (Harvard Apparatus, March, Germany) during the experiment. A laser-Doppler flow probe (PERIMED, PF 5010 LDPM Unit, Sweden) was attached to the right side of the dorsal surface of the skull to monitor regional cerebral blood flow (rCBF). The right common and external carotid arteries were exposed and ligated. The right middle cerebral artery (MCA) was occluded by inserting a silicon rubber-coated monofilament (Doccol Corporation, MA, USA) from the basal part of the right external carotid artery and advanced cranially into the right internal carotid artery to the point where the right middle cerebral artery branched off from the right internal artery. The success of the MCAO was indicated by an immediate drop in rCBF. After the occlusion of the right MCA for 90 minutes, reperfusion was achieved by withdrawing the suture and reopening the right common carotid artery. Animals were allowed to recover for 24 hours. A 24-point scoring system was used to evaluate sensorimotor deficits as described previously^17^. Six tests (spontaneous activity, symmetry of movement, floor walking, beam walking, symmetry of forelimbs, and climbing wall of wire cage) for motor function and two tests (response to vibrissae touch and reaction to touch on either side of trunk) for sensory function were graded on a scale of 0 to 3 each. Neurological scores were assigned as follows: 0, complete deficit; 1, definite deficit with some function; 2, decreased response or mild deficit; 3, no evidence of deficit/symmetrical responses^23^.

### Brain collection and processing

The brains were collected and processed as described previously^17^. Eighteen mice (n = 6 for each group) were euthanized and exsanguinated. Cortical tissues were isolated from peri-infarct area and contralateral corresponding area. The mitochondria, cytosolic and nuclear fractions from the cortical tissues were isolated using FOCUS SubCell Kit (G-Biosciences) following the manufacturer’s instructions. The protein concentration was measured using a BCA assay (Thermo Scientific). The rest eighteen mice (n = 6 for each group) were anesthetized and perfused transcardially with 1X phosphate-buffered saline (PBS), followed by 4% paraformaldehyde in 0.1 M PBS. The brains were removed, fixed overnight in 4% paraformaldehyde in 0.1 M PBS, dehydrated in a graded series of sugar solutions over the course of 72 hours, then embedded in O.C.T. compound (Fisher Scientific) and quick frozen for 5 minutes in liquid nitrogen. The frozen brains were then cut into 14 μm coronal sections and placed on frost-free slides.

### Nissl staining

To measure the infarct size, sections from eight levels (between 2.9 mm rostral and 4.9 mm caudal to bregma) were selected for Nissl staining^24^. The sections were incubated in 0.01% cresyl violet acetate (Sigma) solution for 14 minutes at 60 °C, rinsed in distilled water, dehydrated in ethanol, cleaned in xylene and covered with xylene based mounting media (VWR). The sections were photographed under 1.0x magnification (Olympus) and evaluated using ImageJ. Complete lack of staining defined the infarct lesion. The infarct size was calculated by integration of area of infarct lesion with the distance between coronal levels and expressed as percentage of total hemispheric volume.

### DNA fragmentation

Cytosol mono- and oligonucleosomes were detected by Cell Death Detection ELISA kit (Roche) to evaluate the apoptosis according to the manufacturer’s instructions. Briefly, the wells were coated with anti-histone antibody and incubated with the isolated cytosol fractions, anti-DNA-peroxidase and the substrate. The absorbance of the reaction mixtures was read at 405 nm and expressed as percentage change to the control without I/R group.

### Neuronal Terminal deoxynucleotidyl transferase *dUTP* nick end labeling (*TUNEL*) staining

Three sections (1.21 mm rostral and 0.23 mm and 1.31 mm caudal to bregma) from each mouse were used for evaluating the neural apoptosis. The sections were washed with 1X PBS, blocked with 10% Bovine Serum Albumin (BSA) for at least 1 hour, and then incubated overnight at 4 °C with 1:100 rabbit anti-NeuN (Millipore) for visualization of neuron as primary antibodies. The sections were then incubated with 1:200 AlexaFluor 546 donkey anti-rabbit (Invitrogen) for one hour at room temperature. *In situ* cell death detection Kit (Roche) was used according to the manufacturer’s instruction for TUNEL staining. Briefly, the brain sections were incubated in permeabilisation solution containing 0.1% Triton X-100 and 0.1% sodium citrate for 20 min at room temperature. Then the sections were incubated with a TUNEL reaction mixture for 1 hour at 37 °C, mounted with DAPI mounting medium (Vector) and observed under fluorescence microscope (Olympus). The area with dramatically reduced NeuN staining was defined as the infarct core. For quantitative analysis, both TUNEL and NeuN positive cells were counted in one parietal cortex area and two temporal cortex areas surrounding the infarct core per section and expressed as percentage change to the control group.

### Double immunohistochemical staining

Three sections (1.21 mm rostral and 0.23 mm and 1.31 mm caudal to bregma) from each mouse were washed with 1X PBS, blocked with 10% Bovine Serum Albumin (BSA) for at least 1 hour, and then incubated overnight at 4 °C with mouse anti-NeuN (Millipore) and 1:100 rabbit anti-cleaved caspase-3 (Cell Signaling), or mouse anti-NeuN (Millipore) and rabbit anti-LC3B (Sigma). The sections were then incubated with 1:200 AlexaFluor 546 goat anti-mouse and AlexaFluor 488 goat anti-rabbit (Invitrogen), or 1:200 AlexaFluor 488 rabbit anti-mouse and AlexaFluor 546 donkey anti-rabbit (Invitrogen) for one hour at room temperature. Finally, sections were mounted with DAPI mounting medium with Vector shield and visualized using a Nikon fluorescence microscope (Eclipse Ts2). For quantitative analysis, both NeuN and cleaved caspase-3 or LC3B positive cells were counted in one parietal cortex area and two temporal cortex areas surrounding the infarct core per section and expressed as percentage change to the control group.

### Western blot analysis

Western blot experiments were conducted as described previously^17^. SDS polyacrylamide gel electrophoresis (SDS-PAGE) was performed on a 10% gel on which 20–30 μg of total protein per well was loaded. After SDS-PAGE, the proteins were transferred onto polyvinylidene difluoride membrane. Immunoblotting was performed with the use rabbit anti-Cytochrome C (Cell Signaling), rabbit anti-COX-4 (Cell Signaling), goat anti-AIF (Santa Cruz), mouse anti-HDAC, mouse anti-GAPDH, rabbit anti-LC3B, as primary and peroxidase conjugated goat anti-mouse and goat anti-rabbit IgG as the second antibody. The bound antibody was detected by enhanced chemiluminescence (ECL) detection (Pierce Chemical and Genesee) and the bands were analyzed using ChemiDocTM MP Imaging System (Bio-Rad, CA, USA). For quantification, protein expression was normalized with the loading control and expressed as percentage changes to the control without I/R group.

### Statistical analysis

Data are reported as means ± SE. Differences in infarct size, neurological score, TUNEL-positive neuron, cleaved caspase-3-positive neuron, cytochrome C, AIF, and LC3B-positive neuron between groups were evaluated by one-way ANOVA followed by Dunnett’s test for multiple comparisons to the control group. Differences in DNA fragmentation, LC3B, Bcl-2, and Beclin-1 between groups were evaluated by two-way ANOVA followed by Tukey’s test for significance. A p value of 0.05 or less was considered to be significant.

## Results

### Control conditions

Similar as our previous results^17^, eight weeks of daily feeding with either 0.7 or 2.8 g/kg/day ethanol did not significantly change body weight, mean arterial blood pressure (MABP), heart rate, and fasting glucose level when compared to the control group (Table 1).

**Table 1:** Effect of chronic alcohol consumption on physiological parameters.

	Vehicle	0.7 g/kg/day	2.8 g/kg/d
Body Weight (g)	27.5 ± 0.4	27.2 ± 0.5	26.3 ± 0.4
MABP (mmHg)	85 ± 3	83 ± 4	87 ± 3
Heart Rate (bpm)	577 ± 19	597 ± 43	583 ± 26
Fasting Blood Glucose (mg/dl)	142 ± 7	134 ± 9	147 ± 11

### Effect of chronic alcohol consumption on cerebral *I/R injury*

**Figure 1:**
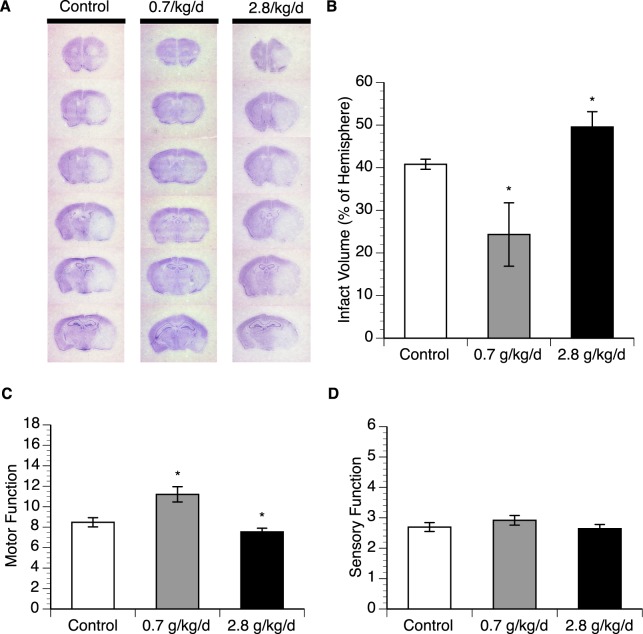
Effect of chronic ethanol consumption on cerebral I/R injury. (**A**) Representative brain sections stained with cresyl violet. (**B**) Total infarct volume. (**C**) Neurological deficit score. Values are means ± SE for 6 mice in each group. *P < 0.05 vs. Control. Analyzed using one-way ANOVA with Dunnett’s post-hoc.

Eight-week consumption of 0.7 g/kg/day ethanol prior to experimental ischemic stroke resulted in a significant decrease (40%) of the infarct volume and markedly improved motor function at 24 hours of reperfusion (Fig. 1). In contrast, eight-week consumption of 2.8 g/kg/day ethanol produced a significant increase (22%) of the infarct volume and exacerbated motor deficits at 24 hours of reperfusion (Fig. 1). However, both doses did not significantly alter sensory deficits at 24 hours of reperfusion (Fig. 1).

### Effect of chronic alcohol consumption on DNA fragmentation

**Figure 2:**
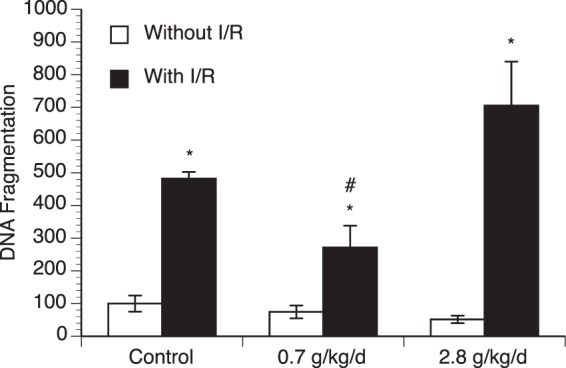
Effect of chronic ethanol consumption on DNA fragmentation in the cerebral cortex. Values are means ± SE for 6 mice in each group. *P < 0.05 vs Without I/R. #P < 0.05 vs Control with I/R. Analyzed using two-way ANOVA with Tukey’s post-hoc.

Measurement of DNA fragmentation was preferentially used to evaluate apoptosis. Eight-week consumption of either 0.7 or 2.8 g/kg/day ethanol did not significantly alter baseline DNA fragmentation. A 90-minute MCAO/24-hour reperfusion produced an increase in DNA fragmentation in all groups. However, the magnitude of the increase was significantly less in 0.7 g/kg/day ethanol group compared to the control group (Fig. 2 and supplementary information).

### Effect of chronic alcohol consumption on apoptosis

**Figure 3:**
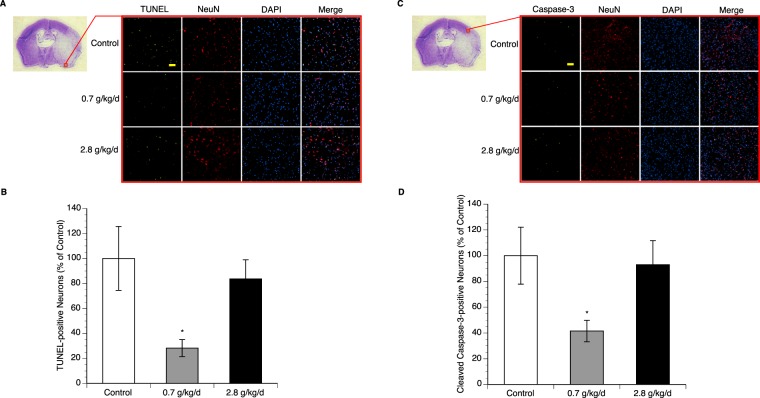
Effect of chronic ethanol consumption on neuronal apoptosis in the cerebral cortex following transient focal cerebral ischemia. (**A**) Representative double staining (10×, scale bar 100 μm) of NeuN and TUNEL in peri-infarct cortex of the temporal lobe at the section 0.23 mm caudal to bregma. (**B**) Values of TUNEL-positive neuron are means ± SE for 6 mice in each group. (**C**) Representative double staining (10×, scale bar 100 μm) of NeuN and cleaved caspase-3 in peri-infarct cortex of the parietal lobe at the section 0.23 mm caudal to bregma. (**D**) Values of cleaved caspase-3-positive neuron are means ± SE for 6 mice in each group. *P < 0.05 vs. Control. Analyzed using one-way ANOVA with Dunnett’s post-hoc.

**Figure 4:**
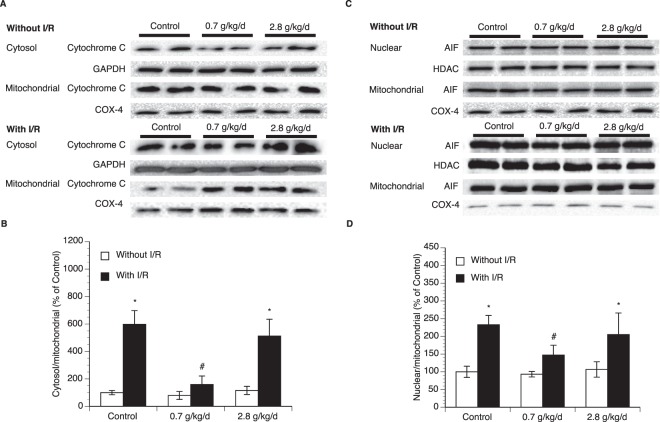
Effect of chronic ethanol consumption on translocation of mitochondrial cytochrome c and AIF in the cerebral cortex following transient focal cerebral ischemia. (**A**) Representative Western blots of cytochrome c (**B**) Values of cytochrome c translocation are means ± SE for 6 mice in each group. (**C**) Representative Western blots of AIF. (**D**) Values of AIF translocation are means ± SE for 6 mice in each group. *P < 0.05 vs. Without I/R. #P < 0.05 vs. Control with I/R. Analyzed using two-way ANOVA with Tukey’s post-hoc.

To examine neuronal apoptosis, double staining of NeuN and TUNEL/cleaved caspase-3 was performed. Eight-week consumption of either 0.7 or 2.8 g/kg/day ethanol did not produce TUNEL-positive and cleaved caspsase-3-positive neuron in the brain before ischemic stroke (data not shown). A 90-minute MCAO/24-hour reperfusion induced TUNEL-positive and cleaved caspsase-3-positive neuron in all groups. However, the number of either TUNEL-positive or cleaved caspsase-3-positive neuron in peri-infarct cortex was significantly less in 0.7 g/kg/day ethanol group compared to the control group (Fig. 3). To determine the potential mechanism, translocation of mitochondrial cytochrome c and AIF was measured. As shown in Fig. 4 and supplementary information, cytosolic translocation of cytochrome c and nuclear translocation of AIF from mitochondria were significantly suppressed in 0.7 g/kg/day ethanol group compared to the control group. In contrast, eight-week consumption of 2.8 g/kg/day ethanol did not alter post-ischemic neuronal apoptosis and translocation of mitochondrial cytochrome c and AIF (Figs. 3 and 4).

### Effect of chronic alcohol consumption on autophagy

**Figure 5:**
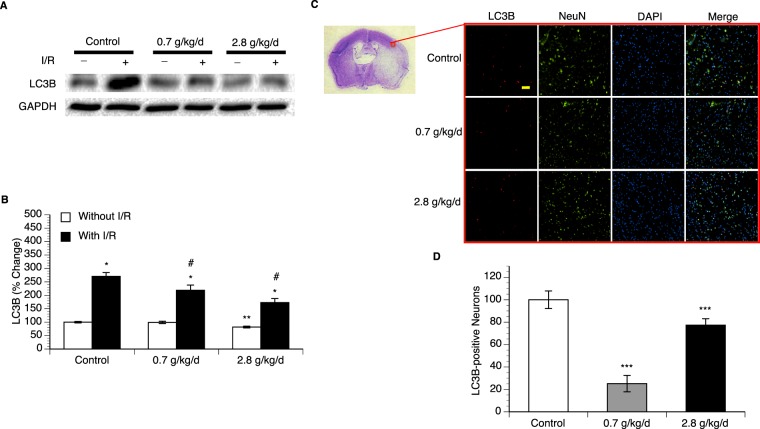
Effect of chronic ethanol consumption on LC3B expression in the cerebral cortex. (**A**) Representative Western blots. (**B**) Values of LC3B expression are means ± SE for 6 mice in each group. *P < 0.05 vs Without I/R. **P < 0.05 vs Control without I/R. #P < 0.05 vs Control with I/R. Analyzed using two-way ANOVA with Tukey’s post-hoc. Analyzed using two-way ANOVA with Tukey’s post-hoc. (**C**) Representative double staining (10×, scale bar 100 μm) of NeuN and LC3B in peri-infarct cortex of the parietal cortex at the coronal section 0.23 mm caudal to bregma. (**B**) Values of LC3B-positive neuron are means ± SE for 6 mice in each group. ***P < 0.05 vs Control. Analyzed using one-way ANOVA with Dunnett’s post-hoc.

Measurement of LC3B expression was used to evaluate autophagy. Eight-week consumption of 2.8 g/kg/day ethanol significantly downregulated expression of LC3B in the cerebral cortex. A 90-minute MCAO/24-hour reperfusion upregulated LC3B expression in all groups. However, the magnitude of the upregulation was significantly less in both 0.7 and 2.8 g/kg/day ethanol groups compared the control group (Fig. 5 and supplementary information). To examine neuronal autophagy, double staining of NeuN and LC3B was performed. No LC3B-positive neuron was detected in all groups before ischemic stroke (data not shown). A 90-minute MCAO/24-hour reperfusion produced LC3B-positive neuron in all groups. However, the number of LC3B-positive neuron in peri-infarct cortex was significantly less in both 0.7 and 2.8 g/kg/day ethanol groups compared to the control group (Fig. 5).

### Effect of chronic alcohol consumption on expression of *Bcl-2* and *Beclin-1*

**Figure 6:**
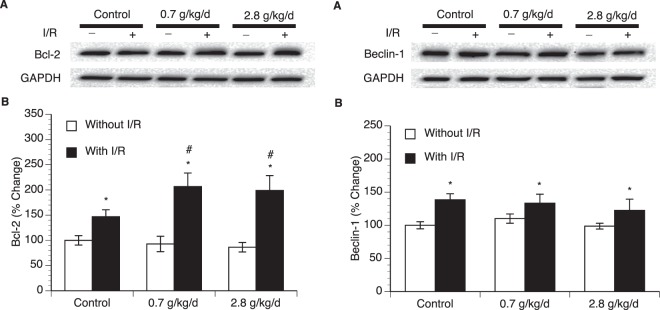
Effect of chronic ethanol consumption on expression of Bcl-2 and Beclin-1 in the cerebral cortex. (**A**) Representative Western blots of Bcl-2. (**B**) Values of Bcl-2 expression are means ± SE for 6 mice in each group. (**C**) Representative Western blots of Beclin-1. (**D**) Values of Beclin-1 expression are means ± SE for 6 mice in each group. *P < 0.05 vs. Without I/R. ^#^P < 0.05 vs Control with I/R. Analyzed using two-way ANOVA with Tukey’s post-hoc.

To determine potential mechanism underlying the influence of ethanol on post-ischemic apoptosis and autophagy, expression of Bcl-2 and Beclin-1 in the cerebral cortex was measured. Eight-week consumption of either 0.7 or 2.8 g/kg/day ethanol did not significantly alter baseline expression of Bcl-2 and Beclin-1 (Fig. 6 and supplementary information). A 90-minute MCAO/24-hour reperfusion significantly upregulated both Bcl-2 and Beclin-1 at 24 hours of reperfusion in all groups. Although the magnitude of upregulation in Beclin-1 was not significantly altered among the groups, the magnitude of upregulation in Bcl-2 was significantly greater in both 0.7 and 2.8 g/kg/day ethanol groups compared to the control group (Fig. 6).

## Discussion

In the present study, the influence of chronic consumption of low and high doses of ethanol on early post-ischemic apoptosis and autophagy of peri-infarct cerebral cortex was investigated. There are three new findings. First, low-dose ethanol alleviated post-ischemic DNA fragmentation and neuronal apoptosis. Second, although chronic consumption of high-dose ethanol worsened cerebral I/R injury, it did not exacerbate post-ischemic DNA fragmentation and neuronal apoptosis. Third, both low-dose and high-dose ethanol consumption attenuated post-ischemic neuronal autophagy. We speculate that chronic consumption of low-dose ethanol may lead to a neuroprotective consequence against cerebral I/R injury by alleviating early post-ischemic apoptosis. On the other hand, chronic consumption of high-dose ethanol may exacerbate cerebral I/R injury by suppressing early post-ischemic autophagy.

Based on our recent experimental results, two doses of ethanol at 0.7 g/kg and 2.8 g/kg were selected to study the dose-dependent influences of ethanol on the cerebral I/R injury in the present study^17^. Gavage feeding with 0.7 g/kg ethanol produced a peak blood ethanol concentration of 9 mM, which is commonly observed in a male person with average body weight (70 kg) following intake of one and a half American standard drinks (14 grams of ethanol/each)^25^. Similar as our previous results^17^, such a low-dose of ethanol significantly decreased infarct size and neurological deficits. On the other hand, gavage feeding with 2.8 g/kg ethanol resulted in a peak blood ethanol concentration of 37.0 mM, which is commonly found in a male person with average body weight following consumption of a little more than seven American standard drinks^23^. Surprisingly, although 2.8 g/kg ethanol significantly acerbated cerebral I/R injury, it did not increase post-ischemic apoptosis. We previously have found that chronic consumption of heavy ethanol worsens cerebral I/R injury by augmenting NMDA-mediated excitotoxicity and NAD(P)H oxidase-mediated oxidative stress^26,27^. Recently, we found that 2.8 g/kg ethanol significantly worsened post-ischemic neutrophil infiltration, microglial activation, and blood-brain barrier (BBB) breakdown^17^. Thus, chronic consumption of high-dose ethanol may exacerbate cerebral I/R injury by augmenting excitotoxicity, oxidative stress, and inflammation but not apoptosis. However, since ethanol affect cerebral I/R injury in a dose-dependent manner, we cannot rule out the possibility that an increased apoptosis may be also involved in the detrimental impact of ethanol at a higher dose.

Apoptosis plays a crucial role in a large number of diseases including neurodegenerative diseases, heart diseases, infectious diseases, autoimmune diseases, cancers, and acute central nervous system insults^28^. Evidence suggests that chronic consumption of heavy ethanol makes cell more prone to undergoing apoptosis^29^. In the brain, there appears to be a regional difference in susceptibility to ethanol-induced damage. The frontal cortex, hippocampus, cerebellum and white matter have been reported to be particularly vulnerable to ethanol-induced apoptosis^30,31^. In addition, it has been generally agreed that ethanol activates the intrinsic apoptotic pathway^29^. However, a recent study found a dose-dependent influence of ethanol on baseline apoptosis in the heart^21^. In contrast to high-dose ethanol, low-dose ethanol actually reduced apoptosis in the heart. In the present study, either 0.7 or 2.8 g/kg ethanol did not produce TUNEL-positive or cleaved caspase-3-positive neuron in the cerebral cortex under basal conditions. Moreover, baseline translocation of mitochondrial cytochrome c and AIF was not altered in either 0.7 or 2.8 g/kg ethanol group compared to the control group. Thus, our findings further suggest that there is a regional difference in susceptibility to anti-apoptotic effect of low-dose ethanol and pro-apoptotic effect of high-dose ethanol. In addition, it is conceivable that the anti-apoptotic effect of low-dose ethanol against ischemic stroke is not related to its effect on baseline apoptosis.

In addition to apoptosis, autophagy also plays a significant role in many diseases, such as metabolic diseases, cardiovascular diseases, infectious diseases, cancer, and neurodegenerative diseases^32^. Interestingly, autophagy plays different roles in different diseases, even in different stages of a disease^32^. Investigators have shown that chronic consumption of heavy ethanol suppresses autophagy in the liver^22,33^. Moreover, suppressed autophagy maybe an underlying mechanism of ethanol-induced liver injury^22^. In the present study, we found that eight-week consumption of 2.8 g/kg/day ethanol significantly reduced expression of autophagosome marker, LC3B, in the cerebral cortex. Basal autophagy has been considered a “housekeeping” process. Many studies have shown that either the absence or inadequate autophagy may be the cause of neurodegenerative diseases^34^. Thus, we speculate that the suppressed autophagy during heavy ethanol consumption may have important implications for the pathogenesis of neurodegenerative diseases observed in chronic alcoholism. On the other hand, although it is generally agreed that autophagy is activated in brain tissue upon ischemic stroke, whether it is a friend or a foe still remains unclear. Its role in ischemic brain damage appears dependent on the degree of autophagy induction and the duration of autophagy activation^5,8^. Excessive and prolonged autophagy is more likely to be detrimental^35^. In the present study, we found that post-ischemic autophagy was reduced at early-stage in both low-dose and high-dose ethanol groups. Since a reduced brain injury was concurrently found in low-dose ethanol group following ischemic stroke, it is conceivable that the reduced autophagy may be reflection of reduced brain injury in the low-dose ethanol group. In contrast, high-dose ethanol produced an exacerbated ischemic brain damage. At least, two possibilities exist. First, it is possible that the reduced autophagy may be an underlying mechanism of increased ischemic brain damage during heavy ethanol consumption. Second, it is possible that the adaptive process of autophagy may be interrupted by the exacerbated ischemic brain damage during heavy ethanol consumption. Thus, the precise role for a reduction in early-stage autophagy in ischemic brain damage during heavy ethanol consumption remains to be determined.

To further determine possible cellular mechanism underlying the altered post-ischemic apoptosis and autophagy, we elected to measure expression of Bcl-2 and Beclin-1. Bcl-2 is localized on the outer membrane of mitochondria, where it prevents cells from undergoing apoptosis by inhibiting the intrinsic pathway^36,37^. In addition, Bcl-2 also inhibits autophagy by interacting with Beclin-1, which is an essential initiator of autophagy^36^. Findings regarding the effect of ischemic stroke on Bcl-2 expression are controversial. Zhang *et al*. found an increase in Bcl-2 expression at 24 hours following cerebral hypoxia-ischemia in mice^38^. Greco *et al*. reported that Bcl-2 expression was upregulated in the cerebral cortex at 5 hours of reperfusion following a 2-hour MCAO in the rat^39^. In contrast, other investigators have reported that Bcl-2 was downregulated at 24 hours of reperfusion^40,41^. In the present study, we found that Bcl-2 protein level in peri-infarct cortex was significantly upregulated at 24 hours of reperfusion. The reason for the discrepancies between these studies are not entirely clear but may be related to the length of ischemia and area of the brain examined. On the other hand, our results regarding the effect of ischemic stroke on Beclin-1 expression are in agreement with the previous findings^42,43^. In the present study, although post-ischemic Beclin-1 was not altered in any ethanol group, post-ischemic Bcl-2 was significantly greater in both 0.7 and 2.8 g/kg/day ethanol groups. Thus, we speculate that reduced apoptosis and autophagy following ischemic stroke in ethanol groups may be related to an increased Bcl-2.

In summary, the present study was the first to determine the influences of both low-dose and high-dose ethanol on apoptosis and autophagy following transient focal cerebral ischemia. We found that a reduced apoptosis may be involved in the neuroprotective effect of low-dose ethanol, whereas a suppressed autophage may be involved in the detrimental effect of high-dose ethanol. Therefore, chronic ethanol consumption appears an important factor implicated in the pathophysiology of transient focal cerebral ischemia. It needs to be considered that therapeutic strategies for treating ischemic stroke in ethanol users should be differed from those in non-ethanol users.

## Supplementary information

Supplementary information.
