# Supplementary material for: Influence of Chronic Ethanol Consumption on Apoptosis and Autophagy Following Transient Focal Cerebral Ischemia in Male Mice
Source: Sci Rep. 2020 Apr 9;10:6164. doi: 10.1038/s41598-020-63213-2 (PMC7145844; doi:10.1038/s41598-020-63213-2)

Influence of Chronic Ethanol Consumption on Apoptosis and Autophagy Following Transient Focal Cerebral Ischemia in Male Mice

Chun Li, Jiyu Li, Guodong Xu, and Hong Sun

Western Blots of Without I/R of Figure 4

Cytosol cytochrome C

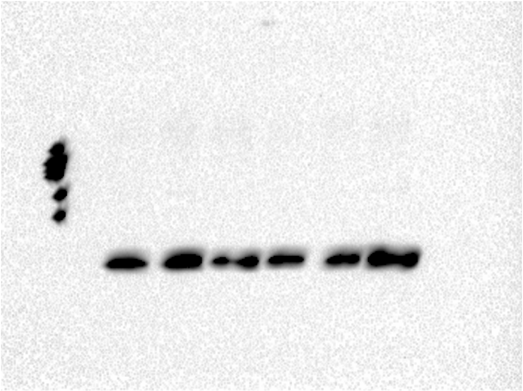

GAPDH

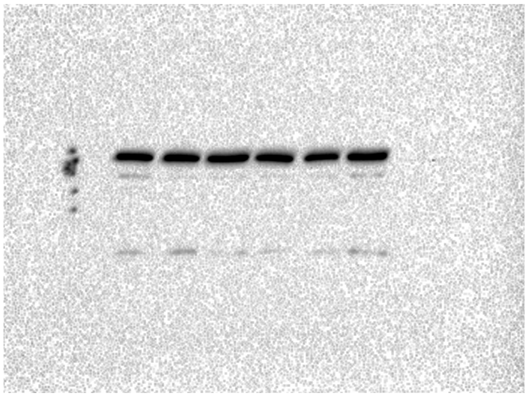

Mitochondrial cytochrome C

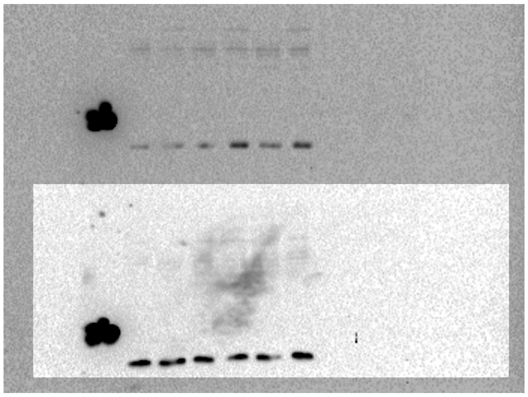

Mitochondrial AIF

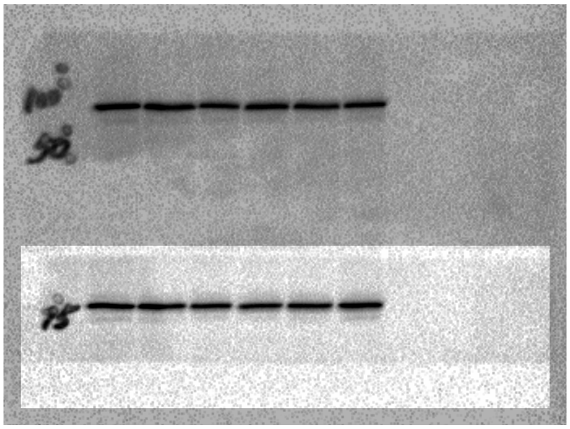

Mitochondrial COX-4

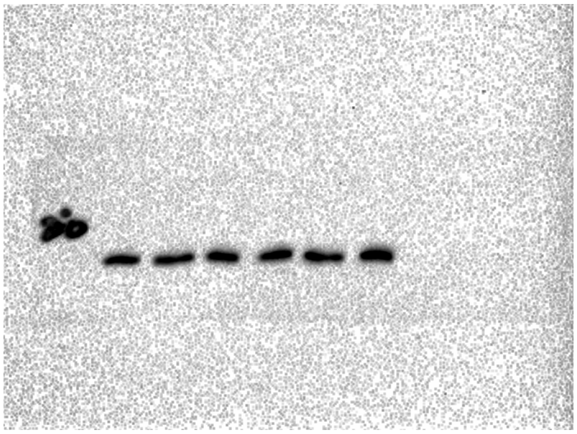

Nuclear AIF

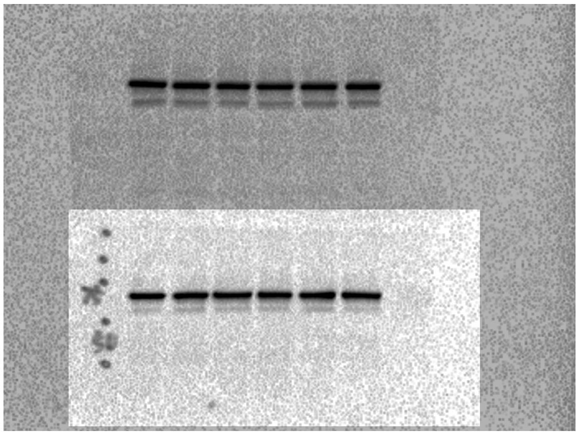

Nuclear HDAC

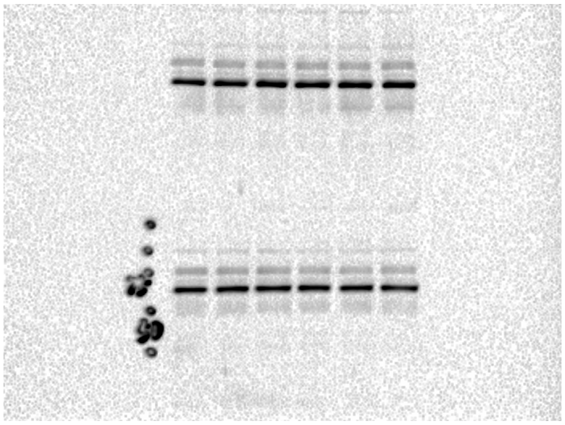

Western Blots of With I/R of Figure 4

Cytosol Cytochrome C and GAPDH

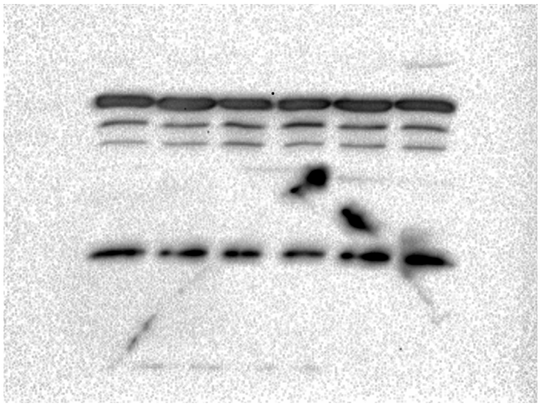

Mitochondrial cytochrome C

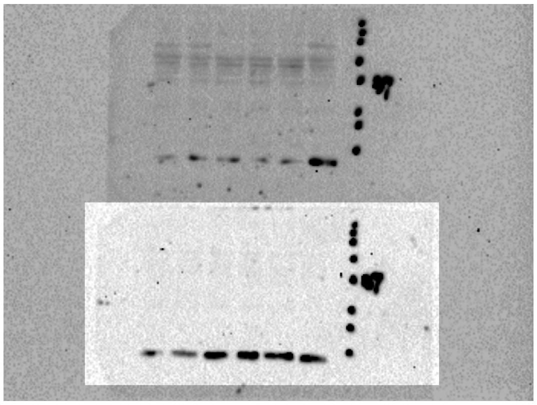

Mitochondrial COX-4

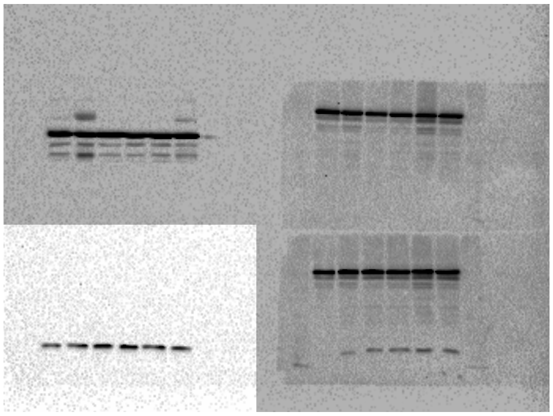

Nuclear AIF

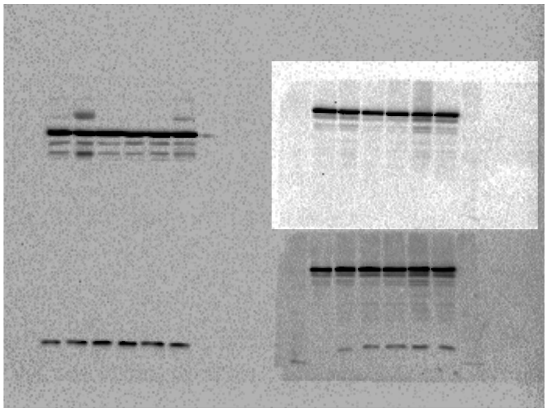

Nuclear HDAC

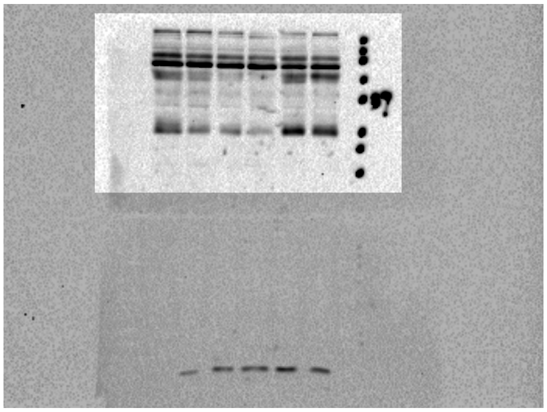

Mitochondrial AIF and COX-4

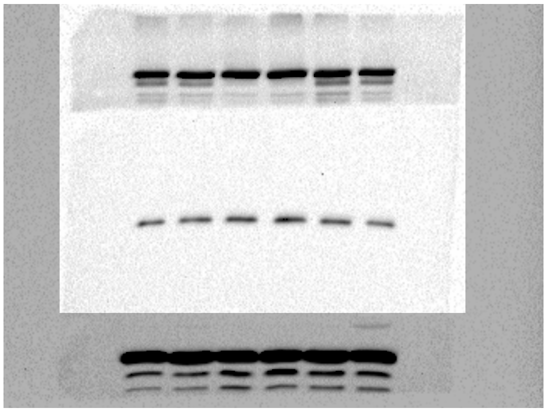

Western Blots of Figure 5

GAPDH and LC3B

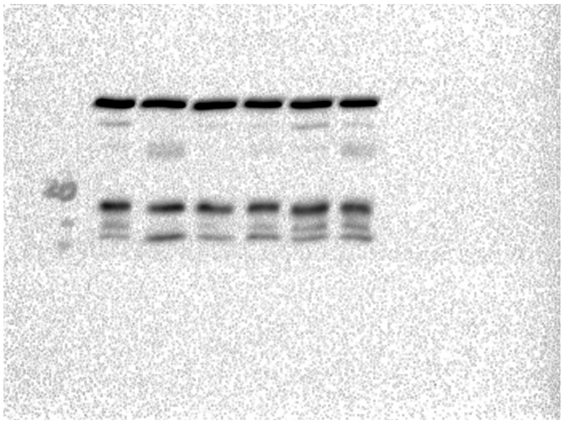

LC3B

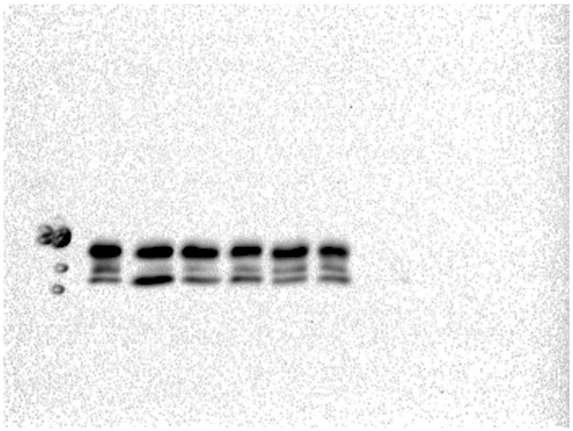

Western Blots of Figure 6.

Bcl-2

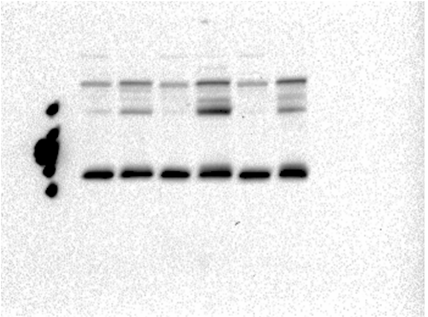

GAPDH

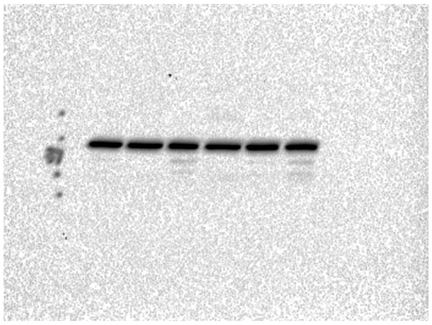

Beclin-1

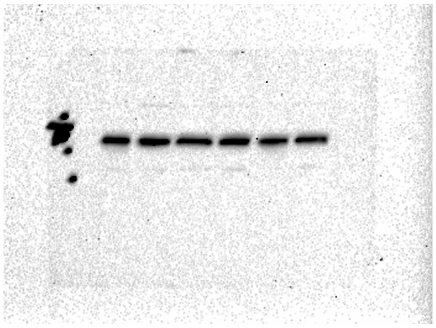

GAPDH

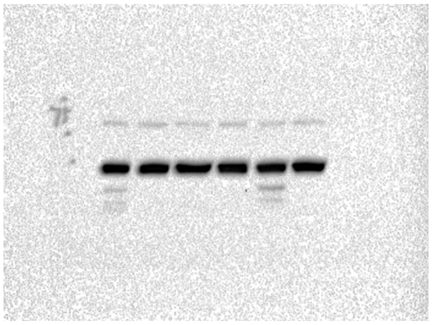

User: USER
Path: C:\Program Files (x86)\BMG\Omega\User\Data\
Test ID: 502
Test Name: Elisa 450
Date: 5/25/2018
Time: 3:41:06 PM
ID1: IL1Beta 11.15.16
Absorbance Absorbance values are displayed as OD

0.091

1. Raw Data (405 1)

	1	2	3	4	5	6	7	8	9	10	11	12
A		0.167	0.309	0.287	0.324	0.141	0.405					
B		1.034	0.983	0.859	0.896	0.899	1.092					
C		0.131	0.152	0.198	0.26	0.29	0.334					
D		0.357	0.372	0.308	0.707	0.81	0.971					
E		0.129	0.158	0.214	0.182	0.155	0.268					
F		1.147	0.776	1.044	1.759	1.089	2.387					
G				0.089								
H				0.093								

2. Raw Data (490 2)

	1	2	3	4	5	6	7	8	9	10	11	12
A		0.042	0.052	0.052	0.055	0.038						
B		0.088	0.094	0.1	0.103	0.103						
C		0.041	0.037	0.043	0.047	0.051						
D		0.056	0.058	0.052	0.085	0.095						
E		0.036	0.039	0.061	0.039							
F		0.123	0.092	0.117	0.173							
G				0.033								
H				0.037								

	0.076	0.218	0.196	0.233	0.05	0.314
	0.943	0.892	0.768	0.805	0.808	1.001
	0.04	0.061	0.107	0.169	0.199	0.243
	0.266	0.281	0.217	0.616	0.719	0.88
	0.038	0.067	0.123	0.091	0.064	0.177
	1.056	0.685	0.953	1.668	0.998	2.296
C	CC	0.1812	0.0409			
	CI	0.8695	0.0372			
	1/2EC	0.1365	0.0328			
	1/2EI	0.4965	0.1138			
	2EC	0.0933	0.0204			
	2EI	1.276	0.2431			

100	22.573
479.94	20.516
75.345	18.08
274.06	62.818
51.518	11.268
704.32	134.2

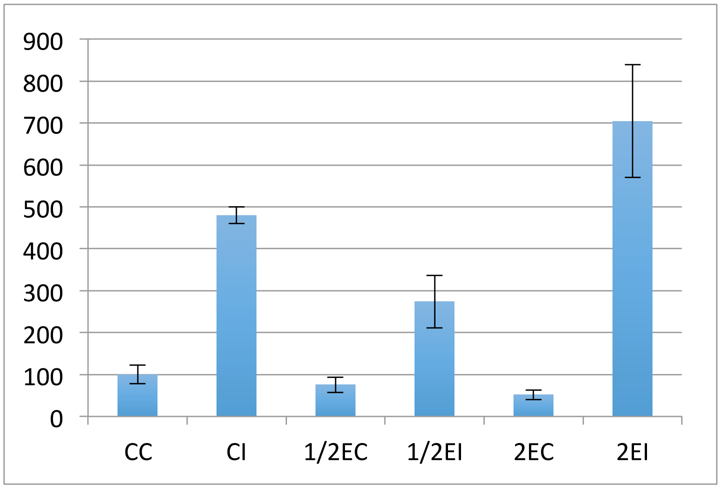

Supplement: Supplementary file 1 — Supplementary information. [file 41598_2020_63213_MOESM1_ESM.pdf]
